# Identification of Reproductive Education Needs of Infertile Clients Undergoing Assisted Reproduction Treatment Using Assessments of Their Knowledge and Attitude

**DOI:** 10.22074/ijfs.2016.4728

**Published:** 2016-11-11

**Authors:** Zahra Ezabadi, Fahimeh Mollaahmadi, Maryam Mohammadi, Reza Omani Samani, Samira Vesali

**Affiliations:** 1 Department of Epidemiology and Reproductive Health, Reproductive Epidemiology Research Center, Royan Institute for Reproductive Biomedicine, ACECR, Tehran, Iran; 2 Department of Endocrinology and Female Infertility, Reproductive Biomedicine Research Center, Royan Institute for Reproductive Biomedicine, ACECR, Tehran, Iran

**Keywords:** Education, Training, Knowledge, Attitude, Infertility

## Abstract

**Background:**

In order to empower infertile individuals and provide high quality patient-centered infertility care, it is necessary to recognize and meet infertile individuals’ educational needs. This study aims to examine infertility patients’ knowledge and subsequently their education needs given their attitudinal approach to infertility education in terms of patients who undergo assisted reproduction treatment.

**Materials and Methods:**

This descriptive study enrolled 150 subjects by conveni- ence sampling of all patients who received their first assisted reproductive treatment between July and September 2015 at a referral fertility clinic, Royan Institute, Tehran, Iran. We used a questionnaire that measured fertility and infertility information (8 questions) as well as attitude toward education on the causes and treatment of infertility (5 questions). Chi-square, independent sample t test, and one way ANOVA analyses were conducted to examine differences by sex. P<0.05 was considered statistically significant.

**Results:**

Total mean knowledge was 3.08 ± 0.99. Clients’ responses indicated that the highest mean knowledge scores related to knowledge of factors that affected pregnancy (3.97 ± 1.11) and infertility treatment (3.97 ± 1.16). The lowest mean knowledge scores related to knowledge of the natural reproductive cycle (2.96 ± 1.12) and anatomy of the genital organs (2.94 ± 1.16). Most females (92.1%) and males (83.3%) were of the opinion that infertility education programs should include causes of infertility and types of treatment associated with diagnostic and laboratory procedures. No statistically significant difference existed between male and female participants (P=0.245).

**Conclusion:**

Most participants in this study expressed awareness of factors that affect pregnancy and infertility treatment. It is imperative to educate and empower infertile individuals who seek reproduction treatment in terms of infertility causes and types of treatment, as well as diagnostic and laboratory procedures to enable them to make informed decisions about their assisted reproductive procedures.

## Introduction

One of the key responsibilities of health care providers is recognizing and meeting patients’ educational needs. It is not only considered as fundamental to patient or client empowerment, but can also promote standards of care and provide high quality patient-centered care (1, 2). Satisfaction with care can originate from adequate patient education, which enables patients to give greater understanding of and participation in medical decision making which often result in better health outcomes (2-4). Bennett et al. (2) have reported that a demand existed for further knowledge in 87% of 212 infertile Indonesian female patients about the causes and treatment of infertility. This finding underlines the need and importance of patient education within the infertility field, especially in developing countries (5, 6). Although there is an enormous gap in educational needs of infertile patients in infertility care centers within resource poor settings, many attempts have been made to investigate knowledge and awareness, in addition to attitude and experiences regarding infertility among various populations (7-9). Relatively little is known about infertility education (6, 8), particularly in patients who receive reproductive treatment (2). To the best of our knowledge no study has investigated these needs according to the perspective of the infertile patient. This was the first study that examined the knowledge and educational needs of infertility patients undergoing assisted reproduction treatment, given their attitudinal approach to infertility education.

## Materials and Methods

This descriptive study recruited 150 subjects by convenience sampling of all infertile clients who received assisted reproductive treatment for the first time between July and September 2015 at a referral fertility clinic (Royan Institute, Iran). This referral clinic assesses people from all socio-economic and ethnic backgrounds.

We measured patients’ knowledge of infertility and educational needs with a questionnaire, designed for Iranian context and validated by a group of 18 gynecologists, embryologists, and conducted face validity of the questionnaire. A graphics expert designed the questionnaire’s font and graphics. The final version of the questionnaire developed by researchers comprised the following two constructs. Initially we requested participants to complete the following demographic information: age (years), sex (male or female), education levels (under diploma, diploma, and academic), occupational status (employed or unemployed), and duration of marriage (years). Then, the questionnaire included two domains that pertained to fertility and infertility information (8 questions), in addition to attitude toward education about the causes and treatment of infertility (5 questions). Question types included yes/no; a Likert scale (too little, little, moderate, much, too much) that ranged from 1 to 5 for knowledge assessment; and the choice of one option and a 5-point Likert scale (too little, little, moderate, much, too much) for attitude assessment.

The Ethics Committee of Royan Institute approved the study (code no: EC/1390/1136). All participants received a complete explanation of the research aims prior to the onset of the study. Voluntary completion of the questionnaire was considered as consent. Eligible individuals were assured that their confidentiality and anonymity, as well as their decision to participate in or withdraw from the study would not impact their current or future relationship with the clinic.

### Statistical analysis

Statistical analyses were carried out using the Statistical Package for Social Science (SPSS, version 15.0 for Windows; SPSS, Inc., Chicago, IL). Continuous variables were expressed as mean ± SD and categorical variables as numbers (percentages). We did not compare the knowledge responses (5-point Likert scale; range: 1 to 5) by sex through the Chi-square test for categorical data. Instead, we used the independent samples t test because it is robust when one may encounter ordinal scaled data. The statistical issue was demonstrated by Heeren and D'Agostino, in 1985 as previously explained (10). The mean differences in infertility knowledge between female and male participants were measured with one-way ANOVA. Chi-square tests of independ- ence were used to assess relationships between categorical variables asked from participants for attitudinal approach. P<0.05 was considered sta- tistically significant.

## Results

Participants had a mean age of 30.93 ± 5.56 years. Females comprised 54% of the study population compared to 46% for males. Only 34% had an academic education. Approximately two-thirds were employed. Demographic characteristics of study participants are presented in Table 1.

**Table 1:** Demographic characteristics of study participants (n=150)


Socio-demographic variables		Number	Percentage

Age (Y)	18-26	25	16.67
	27-32	71	47.33
	33-44	52	34.67
	45-75	2	1.33
Sex	Male	69	46
	Female	81	54
Education level	Under diploma	57	38
	Diploma	42	28
	Graduated	51	34
Occupation	Unemployed	57	38
	Employed	93	62


Table 2 lists participants’ total mean knowledge score of fertility and infertility for each item. As shown, factors that affected pregnancy (3.97 ± 1.11) and infertility treatment (3.97 ± 1.16) had the highest mean knowledge scores. Knowledge of the natural reproductive cycle (2.96 ± 1.12) and anatomy of the genital organs (2.94 ± 1.16) had the lowest mean knowledge scores. We determined the total mean knowledge to be 3.08 ± 0.99. 

**Table 2:** A description of knowledge items from study participants (n=150)


		Mean	SD

K1	Natural reproductive cycle	2.96	1.12
K2	Anatomy of the genital organs	2.94	1.17
K3	Diagnostic tests and procedures	3.07	1.18
K4	Diagnostic surgery	3.12	1.31
K5	Factors affecting pregnancy	3.97	1.11
K6	Infertility treatment	3.97	1.16
K7	Success in infertility treatment	3.08	1.07
K8	Effective factors in the successof infertility treatment	3.20	1.05


Range: 1 (minimum) to 5 (maximum).

**Fig.1:**
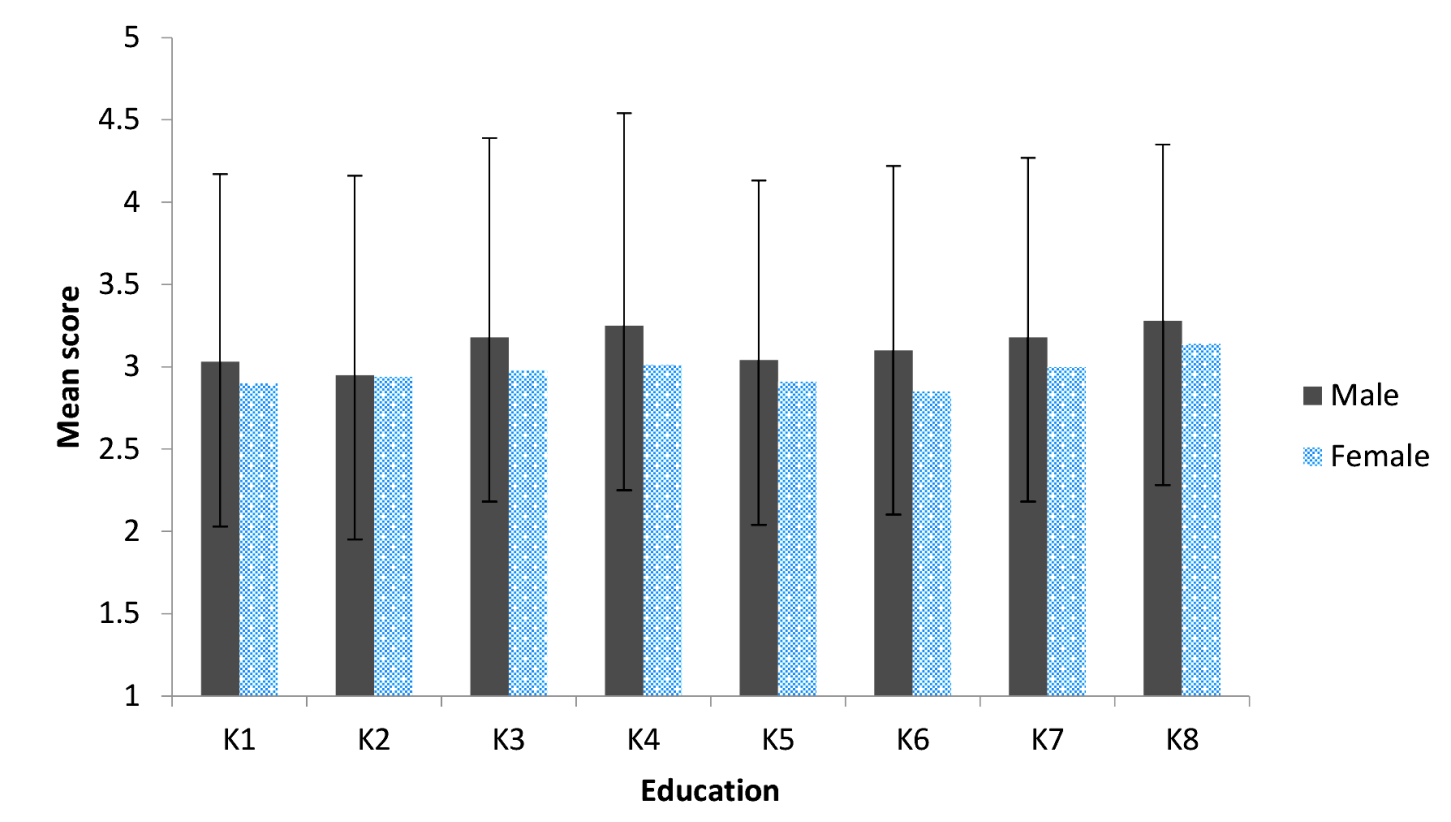
Fertility and infertility knowledge of the 150 respondents by sex. K1; Natural reproductive cycle, K2; Anatomy of the genital organs, K3; Diagnostic tests and procedures, K4; Diagnostic surgery, K5; Factors affecting pregnancy, K6; Infertility treatment, K7; Success in infertility treatment, and K8; Effective factors in the success of infertility treatment. Range: 1 (minimum) to 5 (maximum).

As seen in Figure 1, the highest mean knowledge scores according to gender showed that males scored 3.28 ± 1.07, whereas females had a score of 3.14 ± 1.04 in the question that pertained to effective factors in the success of infertility treatment. This was a nonsignificant difference between males and females (P=0.444). A question on diagnostic surgery showed greater mean knowledge scores of 3.25 ± 1.29 (males) and 3.01 ± 1.32 (females), which was not statistically significant between male and female responders (P=0.266). 

One-way ANOVA showed that infertile participants who had an education level of under diploma had a significantly higher (P=0.042) mean knowledge score of 3.27 ± 1.19 compared to those with diploma (2.80 ± 1.10) and graduates (2.76 ± 1.01). Table 3 lists additional details about fertility and infertility knowledge of the respondents according to level of education. One-way ANOVA showed that infertile participants who had an education level of under diploma had a significantly higher (P=0.042) mean knowledge score of 3.27 ± 1.19 compared to those with diploma (2.80 ± 1.10) and graduates (2.76 ± 1.01). Table 3 lists additional details about fertility and infertility knowledge of the respondents according to level of education. 

Attitude of respondents toward education regarding infertility treatment by sex is presented in Table 4. Of note, approximately 40% of females and 30% males believed in the effectiveness of group education. However 35.4% of males and 25.3% of females preferred individual infertility education. These differences were not statistically significant (P=0.226). Most females (92.1%) and males (83.3%) were of the opinion that infertility education programs should include causes of infertility and types of treatment associated with diagnostic and laboratory procedures. However, no statistically significant difference was found between male and female participants (P=0.245). Views did not differ in terms of the best time for education - whether the first clinic visit or not. The majority thought that education, on average, effectively decreased stress and encouraged cooperation during treatment. Table 5 presents the patients’ attitudes about infertility treatment education in detail. 

**Table 3:** Fertility and infertility knowledge of respondents (n=150) by education


		Education level	Mean	SD	P value

K1	Natural reproductive cycle	Under diploma	3.27	1.19	0.042*
		Diploma	2.80	1.10	
		Graduated	2.76	1.01	
K2	Anatomy of the genital organs	Under diploma	3.15	1.35	0.214
		Diploma	2.73	1.06	
		Graduated	2.90	1.01	
K3	Diagnostic tests and procedures	Under diploma	3.18	1.36	0.586
		Diploma	2.93	0.98	
		Graduated	3.06	1.11	
K4	Diagnostic surgery	Under diploma	3.20	1.47	0.537
		Diploma	2.93	1.27	
		Graduated	3.20	1.15	
K5	Factors affecting pregnancy	Under diploma	3.05	1.21	0.426
		Diploma	2.78	1.01	
		Graduated	3.04	1.06	
K6	Infertility treatment	Under diploma	3.13	1.16	0.346
		Diploma	2.78	1.23	
		Graduated	2.94	1.08	
K7	Success in infertility treatment	Under diploma	3.20	1.12	0.476
		Diploma	2.93	1.15	
		Graduated	3.08	0.96	
K8	Effective factors in the success of infertility treatment	Under diploma	3.32	1.19	0.592
		Diploma	3.13	1.00	
		Graduated	3.14	0.94	


*; P<0.05 was considered statistically significant. Range: 1 (minimum) to 5 (maximum).

**Table 4:** Attitude of respondents (n=150) toward education about infertility treatment by gender


		Group	Male n (%) n=69	Female n (%) n=81	P value

1	Which was the best way to improve awareness of infertility education?	Group	18 (27.7)	32 (40.5)	0.226
		Individual	23 (35.4)	20 (25.3)	
		Does not matter	24 (36.9)	27 (34.2)	
2	Education and counseling should be provided in what context?	Cause of treatment	2 (3.2)	4 (5.1)	0.245
		Type of treatment	1 (1.6)	6 (7.7)	
		Types of treatment cycles	0 (0)	2 (2.6)	
		Diagnostic and laboratorymethods	2 (3.2)	1 (1.3)	
		All items	58 (92.1)	65 (83.3)	
3	When is the best time for the education?	First visit	36 (56.3)	42 (53.2)	0.105
		Before starting treatment	16 (25)	30 (38)	
		During treatment	12 (18.8)	7 (8.9)	
4	How much does education reduce your stress effectively?	Too much	17 (26.6)	21 (26.9)	0.919
		Much	12 (18.8)	18 (23.1)	
		Moderate	17 (26.6)	19 (24.4)	
		Little	7 (10.9)	10 (12.8)	
		Too little	11 (17.2)	10 (12.8)	
5	How much education is effective in your cooperation during the course of treatment?	Too much	19 (30.2)	18 (23.4)	0.491
		Much	11 (17.5)	21 (27.3)	
		Moderate	13 (20.6)	15 (19.5)	
		Little	7 (11.1)	12 (15.6)	
		Too little	13 (20.6)	11 (14.3)	


**Table 5:** Attitude of respondents (n=150) toward education regarding infertility treatment by educational status


		Group	Under Diploma n (%)n=57	Diploma n (%) n=42	Graduated n (%) n=51	P value

1	Which was the best way to improve awareness of infertility education?	Group	20 (37.7)	14 (35)	16 (31.4)	0.408
		Individual	16 (30.2)	8 (20)	19 (37.3)	
		Does not matter	17 (32.1)	18 (45)	31.4 (16)	
2	Education and counseling should be providedin what context?	Cause of treatment	4 (8)	2 (5)	0 (0)	0.181
		Type of treatment	2 (4)	0 (0)	5 (9.8)	
		Types of treatment cycles	0 (0)	1 (2.5)	1 (2)	
		Diagnostic and labo- ratory methods	2 (4)	0 (0)	1 (2)	
		All items	42 (84)	37 (92.5)	44 (86.3)	
3	When is the best time for the education?	First visit	30 (57.7)	18 (45)	30 (58.8)	0.704
		Before starting treat- ment	15 (28.8)	16 (40)	15 (29.4)	
		During treatment	7 (13.5)	6 (15)	6 (11.8)	
4	How much does education reduce your stress effectively?	Too much	10 (19.2)	9 (22.5)	17 (34)	0.079
		Much	7 (13.5)	6 (15)	10 (20)	
		Moderate	14 (27.9)	5 (12.5)	17 (34)	
		Little	9 (17.3)	11 (27.5)	4 (8)	
		Too little	12 (23.1)	9 (22.5)	2 (4)	
5	How much education is effective in your cooperation during the course of treatment?	Too much	11 (22)	6 (15)	18 (36)	0.141
		Much	6 (12)	11 (27.5)	13 (26)	
		Moderate	12 (24)	8 (20)	8 (16)	
		Little	8 (16)	5 (12.5)	8 (16)	
		Too little	13 (26)	10 (25)	3 (6)	


## Discussion

Interestingly, all participants from both sexes gave limited information about their reproductive systems and anatomy of the genital organs. There is scant mention of infertility and sexual health in compulsory secondary school curricula in Iran. From this study, we have determined that males had better knowledge than females on the natural reproductive cycle, diagnostic tests and procedures in infertility, risk factors that affected fertility and pregnancy, and infertility treatment and its success. In contrast, in a survey on infertility knowledge and attitudes in urban high school students, about 20% of students (mostly males) did not recognize that infertility could result from both male and female factors (11). It is well established that awareness of infertility risk factors is essential for fertility preservation (12). Infertility knowledge of male and female risk factors is a critical first step for fertility preservation through lifestyle modification (13-17). Female factor does not always cause infertility, but male factor infertility is responsible of some other cases (15). However, in traditional societies, infertility is known as a female problem (18, 19). 

Research has highlighted that infertility knowledge is associated with education; health promotion strategies are effective when they begin with educational interventions (20). There is an important gap in the literature regarding infertility education. Education about fertility and infertility issues is also needed to prevent fear and unnecessary delay in seeking help and treatment when faced with problems of conception (21, 22). 

The present study was the first to investigate patients’ attitudes toward the effect of education on infertility treatment. Numerous studies aimed to determine knowledge and awareness of infertility among their study population (high school students, medical students, adults, infertile couples, etc.) and to explore an attitudinal trend toward various aspects of infertility (11, 23, 24). On the basis of our results, the majority of women believed group education to be more effective, while most men preferred individual infertility education or neither of the two methods. Therefore, infertility care providers must take this into consideration when designing infertility education. According to the opinion of the vast majority of both sexes, an education program should include causes of infertility and types of treatment associated with diagnostic and laboratory procedures. This education should be conducted at the first visit in order to be more effective in decreasing stress and encouraging cooperation during treatment. Hence, less knowledge about all aspects of infertility, as well as patients’ attitudes toward conditions of infertility education should be taken into account in developing infertility education programs in referral infertility clinics. Very few studies have determined whether public education about infertility is warranted and ultimately effective in prevention. It is recommended that the extent of people’s knowledge of infertility and attitudes about education on infertility should be specified because this would be useful for planning public education programs related to the prevention of infertility, even for the entire society.

The strength of this study was the collection of data on infertile patients’ attitudes toward education on infertility, which thus far has not been considered. Study limitations included the reliance on clients that presented to only one center - a referral clinic for infertility in Iran which limited the generalizability of these findings. This study was cross-sectional and therefore only suggested associations rather than causal relationships. 

## Conclusion

Most participants in this study have expressed awareness of factors that affected pregnancy and infertility treatment. It is imperative to educate and empower infertile individuals seeking reproduction treatment in terms of infertility causes and types of treatment, as well as diagnostic and laboratory procedures in order for them to make informed decisions about assisted reproductive procedures.

Information gathered from this study could be useful for public health educators, health care providers in the clinic, and for government policy makers in order to prepare educational services and programs that meet patients’ needs It seems necessary to provide effective public education on infertility through multiple sources such as media, schools, family, community, health care workers, and the government.
